# Digital cognitive behavioural therapy for insomnia and primary care costs in England: an interrupted time series analysis

**DOI:** 10.3399/BJGPO.2021.0146

**Published:** 2022-03-09

**Authors:** Chris Sampson, Eleanor Bell, Amanda Cole, Christopher B Miller, Tracey Marriott, Matt Williams, James Rose

**Affiliations:** 1 The Office of Health Economics, London, UK; 2 Sleep and Circadian Neuroscience Institute, Nuffield Department of Clinical Neurosciences, University of Oxford, Oxford, UK; 3 Big Health Inc, San Francisco, CA, US; 4 Oxford Academic Health Science Network, The Magdalen Centre, Oxford, UK

**Keywords:** cognitive behavioural therapy, digital technology, healthcare costs, insomnia, interrupted time series analysis, primary health care

## Abstract

**Background:**

*Sleepio* is an automated digital program that delivers digital cognitive behavioural therapy for insomnia (dCBT-I). *Sleepio* has been proven effective in improving sleep difficulties; however, evidence for the possible impact of *Sleepio* use on healthcare costs in the UK has not, to the authors’ knowledge, previously been developed.

**Aim:**

To identify the effect of a population-wide rollout of *Sleepio* in terms of primary care costs in the NHS in England.

**Design & setting:**

The study was conducted in the Thames Valley region of England, where access to *Sleepio* was made freely available to all residents between October 2018 and January 2020. The study relies on a quasi-experimental design, using an interrupted time series (ITS) to compare the trend in primary care costs before and after the rollout of *Sleepio*.

**Method:**

Primary care data for people with relevant characteristics from nine general practices in Buckinghamshire was used. Primary care costs include general practice contacts and prescriptions. Segmented regression analysis was used to estimate primary and secondary outcomes.

**Results:**

For the 10 705 patients included in the sample, the total saving over the 65-week follow-up period was £71 027. This corresponds to £6.64 per person in the sample or around £70.44 per *Sleepio* user. Secondary analyses suggest that savings may be driven primarily by reductions in prescribing.

**Conclusion:**

*Sleepio* rollout reduced primary care costs. National adoption of *Sleepio* may reduce primary care costs by £20 million in the first year. The expected impact on primary care costs in any particular setting will depend on the uptake of *Sleepio*.

## How this fits in

Therapist-delivered cognitive behavioural therapy (CBT) is a recommended first-line treatment for insomnia, but it is difficult to access, and the majority of patients who present for GP management only receive verbal advice or medications for sleep. *Sleepio* has been shown to be clinically effective in 12 randomised controlled trials and offers patients access to fully-automated dCBT-I at scale. The real-world impact of providing whole population access to dCBT-I has not previously been determined in terms of healthcare costs. *Sleepio* was made freely available in a large region of England, and primary care costs were evaluated before and after rollout.

## Introduction

In England, approximately 39% of people have symptoms of insomnia. The prevalence of diagnosed insomnia has increased over time and was 5.8% of the population in 2007.^1^ Insomnia is commonly managed by GPs through verbal advice (100%), sleep hygiene education (89%), and medication.^2^ Patients rarely receive access to first-line CBT for insomnia.^3,4^ This is due in part to a shortage of trained providers^5^ and a lack of treatment awareness.^2,6^ dCBT-I offers a potential solution.

*Sleepio* (https://www.sleepio.com) is a standardised and fully automated (that is, a standalone program without the need for professional human input) digital therapeutic, which comprises the full range of cognitive and behavioural techniques used in CBT for insomnia. It is designed for adults who want to improve their sleep and is available through a website and a supporting app. There is a growing body of evidence to support the effectiveness of *Sleepio*. For example, Espie *et al* ^7^ and Freeman *et al* ^8^ demonstrated benefit in terms of the Sleep Condition Indicator and Insomnia Severity Index. Espie *et al* ^9^ found benefits to functional health, wellbeing, sleep-related quality of life, and symptoms of anxiety and depression.

The societal costs of insomnia are substantial,^10^ and evidence suggests that treatment can be cost-saving.^11,12^ *Sleepio* has been shown to improve workplace productivity,^13,14^ and some evidence indicates that *Sleepio* is associated with reductions in self-reported prescribed sleep medication use.^15^ However, its impact on healthcare service use and prescription costs is mostly unknown. Insomnia is a public health concern, with the costs associated with insufficient sleep estimated to be around £40 billion in the UK owing to lost productivity.^16^ Further costs are thought to be generated from increased healthcare expenditure and accident risk,^11^ with higher healthcare costs for people co-presenting with insomnia and comorbidities.^17^

In England, *Sleepio* could be provided free of charge to the whole population, or to a subset of people who might need it, through NHS funding. Real-world evidence can provide valuable insights beyond evidence generated in a trial setting.

This study sought to address the current gap in knowledge by using real-world data to evaluate the impact of providing public access to *Sleepio* in terms of: 1) primary care service use costs; and 2) prescription costs. The study's main objective was to identify whether providing access to *Sleepio* resulted in a change in the trend of total primary care costs from the perspective of the NHS in England.

## Method

### Intervention

The *Sleepio* program consists of six 20-minute dCBT-I sessions over at least 6 weeks, with each session unlocked 1 week after completing the previous session. People in therapy can maintain a sleep diary to track progress, with advice tailored to the provided information. The sleep diary can be automatically populated with data from a wearable device. Patients can also access other online support tools, including an electronic library and user community.

As part of a 2-year implementation experiment funded by Innovate UK, Big Health Ltd provided access to *Sleepio* in partnership with the Oxford Academic Health Science Network (AHSN).^18^ Population rollout involved all adult residents of the Thames Valley region of England (Oxfordshire, Berkshire, and Buckinghamshire) being granted permission to access *Sleepio* online free of charge. Eligibility was determined by the registrants’s postcode, which must be entered when individuals first use *Sleepio*. Passive promotional activity, such as online and print advertising, was conducted throughout the region to encourage individuals to use *Sleepio*. The largest employers in the region were identified and invited to promote *Sleepio* to their staff and provided with promotional content.

This study gave particular attention to Buckinghamshire, where additional primary care engagement was employed. The *Sleepio* team worked with selected general practices to offer *Sleepio* to patients most likely to benefit from dCBT-I. This work involved evaluating different implementation strategies at population scale and included training, implementing digital prompts for GPs, and providing patient-centred resources to each practice. During the rollout period, additional awareness material was distributed, and practices were given tailored support. This level of engagement was not employed in Oxfordshire and Berkshire.

### Study design

This study adopted a before and after quasi-experimental design. An ITS approach was used to estimate the change in total primary care costs following the rollout of *Sleepio*. The ITS analysis controlled for baseline levels and trends in costs.

The cohort consisted of patients from nine general practices. Patients who were expected to be more likely to use *Sleepio* (based on criteria described below) were selected to reduce noise in the sample.

The study design relies on two key assumptions. First, the primary care costs of people who do not use *Sleepio* are not affected by *Sleepio* rollout. Second, people who do not satisfy the selection criteria do not use *Sleepio*. These assumptions imply that there would be no change in primary care use outside of the sample that is attributable to *Sleepio* rollout.

### Data

EMIS Health is a software company that provides electronic patient record systems to general practices across the UK. Nine GP practices in Buckinghamshire were recruited, from which it was aimed to extract data for ≥10 000 patients from the EMIS system.

Practices were selected to represent a range of levels of deprivation. Patients needed to satisfy at least one of the following criteria within the extraction period:

- a diagnosis of insomnia;
- a diagnosis of depression or anxiety disorder;
- prescription of a hypnotic or anxiolytic medication; or
- referral to *Sleepio* by a GP.

Individuals below the age of 18 years were excluded. The purpose of these criteria was to limit the sample to those who it was anticipated might use *Sleepio*. It was expected that insomnia diagnosis would rarely be coded in the data. Therefore, relevant prescribing (that is, British National Formulary [BNF] section 4.1 hypnotics and anxiolytics drugs^19^) was used as an inclusion criterion to identify people experiencing sleep problems.

Patient-level data were aggregated as patient-weeks, except for time-invariant patient characteristics. The data extraction period was 12 months before *Sleepio* rollout (from October 2017) up to 15 months after *Sleepio* rollout (to January 2020). This provided an adequate timeframe to capture seasonal effects within the ITS design. Notably, the timeframe incorporates three Christmas periods, which were anticipated to be a significant correlate for primary care service use. Data were extracted by an independent specialist provider (Interface Clinical Services Ltd).

Data were also collected from all individual users of *Sleepio*, either through the *Sleepio* website or the supporting iOS app. This sample cannot be linked to the EMIS sample. These data are used for descriptive purposes only, providing information on uptake across the region.

### Analysis

#### Primary analysis

The primary outcome for the analysis is the average primary care costs per patient per week, where primary care costs include GP practice contacts and prescription costs. Unit costs were attributed to resource use using information from the Unit Costs of Health and Social Care.^20^ According to the specific medicine, dose, and pack size, prescription costs were obtained from the BNF Online.^19^

A segmented regression analysis of the ITS data was employed to estimate the change in the trend of the primary outcome, such that the full model was estimated as

where *Y_ijt_* is total primary care costs for individual *i* from practice *j* at time *t*; *time_t_* corresponds with the number of the week in the time series at time *t*; *i* *ntervention*_*t*_ is a binary indicator for time *t* being before (*i* *ntervention* = 0) or after (*intervention* = 1) *Sleepio* rollout; *post_t_* corresponds to the number of weeks after the intervention at time *t*; *X* *_ijt_* is a vector of patient-, practice-, and time-specific confounders; *u_j_* represents a random error term at the level of the practice; and *e_ijt_* represents unexplained variability.

In this model, *ß*_0_ estimates baseline costs at *t* = 0; *ß*_1_ estimates the pre-*Sleepio* trend in costs; *ß*_2_ estimates the immediate change in *Y* at the time of *Sleepio* rollout; and *ß*_3_ estimates the change in trend after *Sleepio* rollout compared with the pre-intervention trend. The post-intervention slope is estimated as *ß*_1_ + *ß*_3_. Seasonal effects are accounted for by including an indicator within *X* for the season in which week *t* falls, where weeks 10–22 of any calendar year are spring, 23–35 are summer, 36–48 are autumn, and 49 through to 9 are winter. *ß*_4_ estimates seasonal and other confounding effects. The rollout period for *Sleepio* was assumed to span 6 weeks (this does not relate to the number of sessions that individuals complete, but rather to the time needed to make *Sleepio* accessible to the whole population).

The segmented regression analysis used a generalised linear model, with appropriate distributions and link functions fitted according to visual inspection of the data and use of a modified Park test and link tests. First-order autocorrelation was tested using the Durban–Watson test. Individual-level observations were drawn from practices, within which observations may be correlated. A multilevel regression model was implemented to account for clustering within practices.

#### Secondary analyses

Four exploratory secondary analyses were conducted, as set out in Table 1. Two of the secondary analyses (A and B) focused on prescriptions. These analyses could help healthcare commissioners to better understand the impact on costs and resource use characterised in the primary analysis. The budgetary implications of changes in prescription costs may differ from those associated with changes in GP contacts. The long-term prescription of non-benzodiazepines (zolpidem and zopiclone, commonly known as Z-drugs) for sleep problems is a concern in and of itself, owing to their side effects and lack of effectiveness.^4^

**Table 1.** Overview of secondary analyses

**Analysis**	**Population**	**Intervention**	**Outcome**
A	People referred to *Sleepio* by their GP	*Sleepio* rollout	Total prescription costs
B	People referred to *Sleepio* by their GP	*Sleepio* rollout	Count of Z-drug prescriptions
C	People with a diagnosis of anxiety or depression	*Sleepio* rollout	Total primary care costs
D	Whole sample	*Sleepio* referral	Total primary care costs

For analyses A and B, only individuals who were referred to *Sleepio* — at any point in the follow-up period — were included. Reducing the size of the dataset in this way made it possible to more effectively control for individual-level variation, and potentially identify effects directly attributable to *Sleepio* use.

Previous research on *Sleepio* has demonstrated benefits in terms of anxiety or depression symptoms and the potential for *Sleepio* to be particularly effective for people with anxiety or depression.^8,9,21^ To contribute to this evidence base, the primary analysis was implemented with stratification according to the presence of a diagnosis of anxiety or depression.

To further test the robustness of the findings and the extent to which any treatment effect is attributable to *Sleepio* rollout, analysis D was conducted whereby referral to *Sleepio* is the intervention. In this case, the control group is the population of patients who were never referred to *Sleepio*. An analysis similar to the primary analysis was implemented, using a hierarchical generalised linear model to evaluate the effect on primary care costs, in any given week, of having been referred to *Sleepio* by a GP. This approach is more susceptible to selection bias, but could provide more precise identification of patients who use *Sleepio* and reduce noise in the sample.

## Results

The analysis included 1 252 485 patient-week observations from 10 705 patients over 117 weeks. Of the sample, 64.41% identified as female. Age was observed in 5-year bands (except for those aged 18–25 and >90 years). The median age band was 50–55 years; 50.21% of the sample were aged 35–65 years (data not shown). Table 2 shows the number of patients in each practice and the number recorded as being referred to *Sleepio* at least once within each practice, of which there were 1008 patients in total.

**Table 2.** GP practice samples

		**EMIS data**	
		**Referred to *Sleepio***	
**Practice**	**Sample size, *n***	***n***	**%**
1	2391	220	9.20
2	812	87	10.71
3	1849	171	9.25
4	295	33	11.19
5	757	64	8.45
6	1511	138	9.13
7	454	79	17.40
8	2351	148	6.30
9	285	68	23.86
Total	10 705	1008	9.42

Across the sample, 1655 (15.46%) people had at least one record of an insomnia diagnosis, and 5515 (51.52%) had at least one diagnosis of anxiety or depression. Inclusion criteria prescriptions (hypnotics or anxiolytics) were received by 3001 (28.03%) people, with 1919 (17.93%) receiving at least one hypnotic prescription and 1502 (14.03%) receiving at least one anxiolytic prescription (data not shown).

In October 2018, the total number of patients registered with the nine practices in the study was 129 865, and the total population of Buckinghamshire was around 540 059.^22^ Table 3 illustrates the estimated number of patients who used *Sleepio* for different population sizes, based on the number of people recorded as being referred by their GP in the EMIS data and the actual number of patients recorded by *Sleepio* within the full 117 weeks of the study.

**Table 3.** Estimated number of patients who used Sleepio by population

		**Estimated patients based on EMIS data**		**Estimated patients based on *Sleepio* data**	
**Population**	**Population size, *n***	***n***	**%**	***n***	**%**
EMIS sample	10 705	1008	9.42	Unknown^a^	Unknown^a^
Nine practices	129 865	1008^b^	0.78^c^	1220	0.94
Buckinghamshire	540 059	4192^c^	0.78^c^	3134	0.58
Thames Valley	2 300 000	17 940^c^	0.78^c^	12 374	0.54
England	55 980 000	434 512^c^	0.78^c^	302 292^c^	0.54^b^

^a^Unknown represents the number of people across the EMIS sample who registered in the Sleepio app, which the authors were not able to observe because the two data sets could not be linked. ^b^Extrapolated from observation. ^c^Assumed from extrapolation.

The EMIS data only included referrals recorded by GPs, while the *Sleepio* data included all referral routes. *Sleepio* data relating to the nine practices rely on patients self-reporting GP referral. An individual’s county is determined by their postcode provided in *Sleepio*. The Thames Valley region was taken as comprising the three counties of Buckinghamshire (including Milton Keynes), Oxfordshire, and Berkshire.

### Primary analysis

The regression results from the primary analysis are shown in Table 4. Several models are reported as a sensitivity analysis of alternative specifications.

**Table 4.** Full sample regression coefficients

Category	**Preferred model**	**Model 2**	**Model 3**	**Model 4**	**Model 5**
Time	0.002^a^	0.002^a^	0.002^a^	0.001^a^	0.004
Intervention	–0.038^a^	–0.036^a^	–0.033^a^	0.033^a^	0.263^a^
Post	–0.002^a^	–0.002^a^	–0.002^a^	–0.000	–0.003
Seasonal adjustment	YES	YES	YES	NO	NO
Age bands	YES	YES	NO	NO	NO
Sex	YES	YES	NO	NO	NO
Diagnoses	YES	NO	NO	NO	NO
Practice random effects	YES	YES	YES	YES	NO

^a^ *P*<0.001.

All models except Model 5 are hierarchical generalised linear models using a quasi-gamma distribution with a variance function of *V* (*µ*) = *µ* ^2^ and log link. Model 5 is an ordinary least squares linear regression model. The preferred model is that which exhibited superiority in statistical tests and provided the most convincing predictions visually.

A positive coefficient for *Time* shows that primary care costs increase over time, before *Sleepio* rollout. A negative coefficient for *Intervention* implies that the immediate impact of *Sleepio* rollout (during the 6-week rollout period) is to reduce primary care costs. A negative coefficient for *Post* shows that the effect of *Sleepio* rollout was to reduce the trend shown by the coefficient on *Time*. If the negative coefficient for *Intervention* is smaller than the positive coefficient for *Time*, it implies that the upward trend in primary care costs continues after *Sleepio* rollout, but at a slower rate.

The regression results imply three key overall findings. First, there is a slight increasing trend in primary care costs over time. Second, *Sleepio* rollout has a small but statistically significant negative impact on costs during the initial 6-week rollout period. Third, *Sleepio* rollout mitigates the trend of increasing primary care costs.

Comparison between the alternative model specifications reveals that seasonal adjustment is critical; accounting for seasonal effects reverses the direction of effect for *Intervention* and results in statistical significance for *Post*.

The preferred model results show that the absolute difference in mean weekly costs per person, associated with *Sleepio* rollout, is a saving of £0.16 at week 65. This corresponds to £6.64 per person over the 65-week follow-up period, including the initial rollout period. The 95% confidence interval (CI) for this estimate is a saving of between £4.60 and £8.67. Across the observed sample of 10 705 people, *Sleepio* rollout reduced primary care costs by £71 027 (95% CI = £49 291 to £92 762) (Table 5).

**Table 5.** Projected reduction in primary care costs associated with Sleepio rollout

**Sample**	**1** year	**65** **weeks**	**2 years^a^**	**3 years^a^**
Per person(95% CI)	£4.66(£3.20 to £6.13)	£6.64(£4.60 to £8.67)	£13.00(£9.15 to £16.84)	£21.48(£15.22 to £27.75)
Per *Sleepio* user^b^	£49.52	£70.44	£138.00	£228.07
Nine practices	£49 930	£71 027	£139 144	£229 967
Buckinghamshire	£207 640	£295 374	£578 648	£956 347
Thames Valley	£884 298	£1 257 936	£2 464 343	£4 072 885
England	£21 523 042	£30 617 071	£59 979 956	£99 130 468

^a^Projection beyond the observed period. ^b^9.42% of the sample based on GP referrals.

Table 5 presents projections for average cost savings for different populations over different durations, based on the preferred model specification. The projections assume that, beyond the observation period (that is, 65 weeks), primary care costs return to the trend observed before *Sleepio* rollout (represented by the coefficient for *Time* in Table 4).

Table 5 includes estimates per *Sleepio* user, based on the uptake estimates in Table 3, which assumes a growing population of users with growth at the rate observed in the study. Economic evaluations and decision analyses often rely on estimating effects at the individual level, which this study does not observe. Therefore, to inform future research, alternative projections are provided based on changes over time at the individual level. These projections are based on the assumption that *Sleepio* rollout is equivalent to treatment exposure at the individual level, such that those people identified as *Sleepio* users become *Sleepio* users at the point of the rollout. These projections assume a return to pre-rollout trends and no new users after Year 1, such that Year 1 savings for projected new users are subtracted from projected cumulative savings. In this case, the 2-year savings per user would be £88.48 (£138.00 minus £49.52). The 3-year savings would be £139.59 (£228.07 minus £88.48). Rather than projecting a growing saving over time as trends diverge, this approach assumes a shift to the pre-rollout trend to converge on an annual saving of £90.07 per user in the long term. Based on existing evidence, these effects might be expected to be maintained for up to 3 years.^23^

### Secondary analyses

Table 6 shows the key coefficients for the secondary analyses that maintained the segmented regression analysis approach, as summarised in Table 1. All analyses included seasonal adjustment, age, sex, diagnoses, and practice random effects. Analysis A and B used a Poisson distribution with a log link. Analysis C used the same model specification as the primary analysis.

**Table 6.** Secondary analysis regression coefficients

	**Analysis A (prescription costs**)	**Analysis B (Z-drugs**)	**Analysis C (anxiety/depression sample**)
Time	0.005^a^	0.002^b^	0.003^a^
Intervention	0.013	0.222^a^	–0.046^a^
Post	–0.004^a^	–0.003^c^	–0.003^a^

^a^ *P*<0.001. ^b^ *P*<0.05. ^c^ *P*<0.01.

The coefficient for the pre-existing increasing trend in prescription costs (0.005) is greater than that for overall costs (0.002), as is the coefficient for the impact of *Sleepio* on the trend (–0.004 compared with –0.002). The coefficients for Analysis A can be used to estimate a reduction in prescription costs at 65-week follow-up of £8.62 per person (£5.40 in the first year), suggesting that the observed reductions in prescription costs of the primary analysis may significantly explain savings. Analysis B shows that *Sleepio* had a small but statistically significant impact in achieving a downward trend in the prescription of Z-drugs. Analysis C supports the notion that *Sleepio* may be more effective in reducing costs among people diagnosed with anxiety or depression (*n* = 5515). The average saving per person in this group, over 65 weeks, was £9.27.

The final analysis (D), treating *Sleepio* referral as the intervention, identified a negative but statistically non-significant effect. This is likely owing to a selection bias, whereby individuals who use *Sleepio* may be more likely to have higher levels of resource use, all else being equal. An exploratory analysis of the insomnia subsample did not identify any statistically significant findings. This may be owing to difficulty with coding insomnia or a lack of power associated with the sample size.

## Discussion

### Summary

The main findings of the analysis show that the rollout of *Sleepio* in the Thames Valley region reduced average primary care costs, including general practice contacts and prescriptions.

Over the observed follow-up period, the average saving in the sample was £6.64 per person. Assuming that people outside of the sample were not affected by the rollout of *Sleepio*, this corresponds to a saving of £71 027 across the nine practices.

The secondary analyses suggest that reductions in prescription costs are a significant driver in reducing overall primary care costs. This is partly explained by reductions in the prescription of Z-drugs, which may include both off-label and on-label use. Data from *Sleepio*, collected separately from the present analysis, suggest that around 26% of people referred to *Sleepio* would start CBT (according to Big Health Ltd operational data for the Thames Valley region). Based on these figures, the saving per patient associated with prescription costs may be £20.77 in the first year (£5.40/0.26), around half of the per-user saving identified across the whole sample.

The reduction in costs observed in the subsample of people diagnosed with anxiety or depression (*n* = 5515) was greater than the average saving across the whole sample. Future research should explore the potential for cost savings in different categories of expenditure and different populations.

### Strengths and limitations

A strength of the analysis is that the primary and secondary outcomes and analytical approach were determined before the data were analysed. The preferred model was selected on the basis of predictive ability and fit for the primary outcome.

The main findings are robust across a variety of model specifications. The observed direction of effect on the trend in costs is not sensitive to seasonal effects or individuals’ characteristics. This supports the generalisability of the findings. Practices with different proportions of people with the diagnoses observed in the data may, therefore, expect to observe similar impacts for the subpopulation that satisfies the inclusion criteria.

The inclusion of seasonal effects and practice random effects was important in demonstrating a statistically significant impact of *Sleepio* rollout. However, the direction of effect for the trend in costs was not undermined by their exclusion. The evidence suggests that the cost savings were not driven by any single practice, supporting the findings‘ generalisability. However, variation was observed by practice, and commissioners should consider the characteristics of providers and patients that may act as barriers or facilitators to changes in service use.^18^

It is important to note that this study is not a cost-effectiveness analysis, and the estimates do not include other potential cost impacts associated with *Sleepio* rollout. In particular, the analysis does not attribute any direct cost to *Sleepio* rollout or use. The advantage of this is that the analysis is independent of *Sleepio* pricing. The relevance of the study will not be undermined by changes in the price of *Sleepio* licences.

In practice, access to *Sleepio* may be priced on the basis of initiation of dCBT-I rather than initial registration by a user or referral by a GP. A limitation of the analysis is that whether individuals in the EMIS data sample initiated dCBT-I cannot be identifed.

It was not possible to describe the sample in detail owing to the limited availability of personally identifiable data, as agreed with participating practices. This makes it difficult to assess the representativeness of the sample. As a result, the extrapolations to the national level are speculative; it is not possible to weight the observations according to the characteristics of the population as a whole, which may be particularly important in considering the uptake of a digital technology.^24^

The study was also unable to observe any changes in resource use in areas outside of Buckinghamshire, where primary care engagement was based on passive promotional activity and not used to drive uptake. As shown in Table 3, a lower level of uptake was observed in these areas. This has implications for implementation strategies and related costs. Further work could evaluate the resource impact of *Sleepio* rollout in alternative settings, such as when delivered as an adjunctive intervention for those with poor sleep through the Improving Access to Psychological Therapies (IAPT) programme. The analysis does not provide estimates of any impact on referrals to secondary care or resources used in settings other than GP practices. Owing to the pre-specification of the primary outcome and the development of the model to suit these data, there was limited scope for the study to explore different aspects of resource use without suffering from overtesting. The design of the analysis also limits the extent to which the impact on resource use relating to GP contacts compared with that associated with prescription costs can be estimated.

Optimal sample specifications for identifying statistically significant effects in ITS analyses are difficult to estimate reliably.^25^ One limitation of this study is that simulations to specify a sample size were not conducted before data collection was commenced. The sample size was determined on the basis of practicality and the authors’ expectations about uptake and the variability in healthcare costs. Nevertheless, the total number of observations is likely to provide reliable estimates.^26^

An alternative study design may have enabled the study to overcome several of the limitations described here. Observation of a contemporaneous comparator, such as practices outside of the rollout area, would have enabled accounting for unobserved causes of variation in healthcare costs. However, it was not possible to establish the necessary contacts and agreements to support such a design.

### Comparison with existing literature

To the authors’ knowledge, no studies have evaluated the real-world healthcare cost impact of providing CBT for insomnia at population scale. Previous health economic evaluations of CBT for insomnia have focused on evaluations using data from controlled trials,^27^ which may not be generalisable to primary care settings where GPs manage insomnia.^2^ This analysis supports previous research showing that dCBT-I can reduce prescribing.^15^

### Implications for practice

The rollout of *Sleepio* in the Thames Valley resulted in lower primary care costs across nine practices within 1 year. Providing NHS patients in England with access to *Sleepio*, while encouraging GPs to refer patients with sleep problems to register for *Sleepio*, is likely to result in fewer GP attendances and fewer prescriptions in the population. Therefore, these savings will partially or entirely offset direct costs associated with the adoption of Sleepio.
